# Molecular Fingerprint of Human Pathological Synoviocytes in Response to Extractive Sulfated and Biofermentative Unsulfated Chondroitins

**DOI:** 10.3390/ijms232415865

**Published:** 2022-12-14

**Authors:** Valentina Vassallo, Antonietta Stellavato, Rosita Russo, Donatella Cimini, Mariangela Valletta, Alberto Alfano, Paolo Vincenzo Pedone, Angela Chambery, Chiara Schiraldi

**Affiliations:** 1 Department of Experimental Medicine, Section of Biotechnology, Medical Histology and Molecular Biology, University of Campania “Luigi Vanvitelli”, 80138 Naples, Italy; 2 Department of Environmental, Biological and Pharmaceutical Sciences and Technologies, University of Campania “Luigi Vanvitelli”, 81100 Caserta, Italy

**Keywords:** extractive chondroitin sulfate, biofermentative unsulfated chondroitin, human primary synoviocytes, intracellular proteomic

## Abstract

Pharma-grade extractive chondroitin sulfate (CS) is widely used for osteoarthritis (OA) treatment. Recently, unsulfated biofermentative chondroitin (BC) proved positive effects in OA in vitro model. This study, based on primary pathological human synoviocytes, aimed to analyze, by a multiplex assay, a panel of OA-related biomarkers in response to short-term treatments with bovine (CS_b_), pig (CS_p_) and fish (CS_f_) chondroitins, in comparison to BC. As expected, all samples had anti-inflammatory properties, however CS_b_, CS_f_ and especially BC affected more cytokines and chemokines. Based on these results and molecular weight similarity, CS_f_ and BC were selected to further explore the synoviocytes’ response. In fact, Western blot analyses showed CS_f_ and BC were comparable, downregulating OA-related biomarkers such as the proteins mTOR, NF-kB, PTX-3 and COMP-2. Proteomic analyses, performed by applying a nano-LC-MS/MS TMT isobaric labelling-based approach, displayed the modulation of both common and distinct molecules to chondroitin treatments. Thus, CS_f_ and BC modulated the biological mediators involved in the inflammation cascade, matrix degradation/remodeling, glycosaminoglycans’ synthesis and cellular homeostasis. This study helps in shedding light on different molecular mechanisms related to OA disease that may be potentially affected not only by animal-source chondroitin sulfate but also by unsulfated biofermentative chondroitin.

## 1. Introduction

Nowadays, some factors such as longevity and the increase in obesity, have made osteoarthritis (OA) among the most common joint diseases worldwide [1,2,3]. OA causes joint pain and stiffness with progressive cartilage matrix degradation and bone sclerosis, leading to chronic disability that affects the patient’s quality of life [4,5]. The scientific community agrees that synovitis is associated with articular pain and OA advancement [6]. Fibroblast-like synoviocytes represent the major cellular component of synovium that produce many biological mediators (nuclear factor kappa-light-chain-enhancer of activated B cells (NF-kB), pro-inflammatory interleukins such as IL-1β and IL-6, matrix metalloproteases (MMPs), tissue inhibitor of metalloproteinases (TIMP-1) and cartilage oligomeric matrix protein (COMP)) affecting immune cells’ activation, vascular hyperplasia and inflammation [7,8,9]. It has been reported that collagen and proteoglycan reduction in the synovial fluid increase the inflammatory signaling cascade with a central role played by cytokines [10,11]. In addition, synoviocytes produce hyaluronic acid (HA), through hyaluronic acid synthase (HAS), which is the major substance responsible for the viscoelastic behavior of the synovial fluid. Besides the presence of other proteoglycans and/or glycosaminoglycans (GAGs), HA concentration and molecular weight affect the composition and rheological properties of pathological synovial fluid [12,13]. Currently, OA treatments are based on the use of both non-pharmacological and pharmacological therapies. The first strategy aims at improving patient lifestyle (i.e., losing weight by exercising), while pharmacological approaches are concentrated on the use of analgesics and non-steroidal anti-inflammatory drugs (NSAIDs) [14,15]. Chondroitin sulfate-based treatments have been reported to be endowed with positive effects on OA-suffering patients [16,17,18,19]. Specifically, chondroitin sulfate (CS) is a natural biomacromolecule present in all vertebrates and invertebrates having a role in numerous biological processes such as support for cell growth, wound healing repair and suppression of pro-inflammatory cytokine activities [20,21,22]. It is a linear polysaccharide composed of repeating disaccharide units (D-glucuronic and N-acetyl-D-galactosamine) and sulfate groups at different positions on sugar residues [23]. This biopolymer can be extracted from different animal sources, thus presenting different purity grades [24] and many studies analyzed the potential biological features of different chondroitin sulfation patterns and extractive sources [24,25,26,27]. The recent literature showed that this biopolymer stimulates the chondrocyte’s production of proteoglycans in vitro, inhibits the expression of pro-inflammatory cytokines and counteracts metalloproteases’ activity preventing cartilage damage. The CS action mechanism is not completely revealed, also considering the heterogeneity of the molecule (source, molecular weight (MW), sulfation pattern), and even more BC-specific features have to be investigated since there was a common opinion asserting that sulfation was directly correlated to bioactivity [25]. The presence of CS is predominant in cartilage tissues with anti-inflammatory and chondroprotective effects [28]. Moreover, unsulfated chondroitin is also present at low concentrations in human connective tissues [29]. Although extensive information about the cellular effects of CS is increasingly growing, a wide molecular fingerprint of effects elicited by unsulfated chondroitin is still lacking. For this reason, to deepen our understanding of pharma-grade CS effectiveness and better explore the properties of biofermentative unsulfated chondroitin in counteracting OA degenerative and inflammatory process, experimental tests were performed exploiting a well-established OA in vitro model based on pathological human synoviocytes [30,31]. Specifically, the unsulfated chondroitin (biofermentative chondroitin (BC)) used in the present study has been obtained through a validated fermentative process, followed by extensive purification procedures [32,33]. The introduction of BC in therapeutic applications could represent a potential solution to overcome issues related to ethical and/or religious concerns regarding both the extraction sources and methods [34]. Furthermore, previous studies showed that BC was more effective than CS on an OA in vitro model based on IL-1β-insulted nasal human chondrocytes [35]. More recently, the secretome of BC-stimulated synoviocytes was compared to the fish CS-stimulated ones, reporting a comparable, or even better effect of BC [31]. In addition, the combination of BC with HA was used to assess the potential anti-inflammatory and restorative effects in both pathological chondrocytes and synoviocytes [30]. In the present work, we first investigated the modulation of cytokines, chemokines and growth factors’ profile of primary pathological synoviocytes in response to four different animal-source chondroitins (bovine CS_b_, pig CS_p_, fish CS_f_ and biotechnological BC) by multiplex immunoassay. All chondroitins used in this experimental set-up have been analyzed for their purity grade and molecular weight distribution [26]. Based on their higher efficacy in inducing modulation of mediators of inflammation, CS_f_ and BC treatments were selected for an in-depth quantitative proteomic analysis aimed at characterizing the response of osteoarthritic synoviocytes to these two specific chondroitin treatments. In this OA in vitro model, CS_f_ and BC confirmed a similar biological effect as previously observed at the extra-cellular level [31]. Our results provide an overview of proteins modulated by treatments, thus improving our knowledge of pathways affected by different chondroitins potentially related to OA disease.

## 2. Results

### 2.1. Bioplex Assay

**Figure 1:**
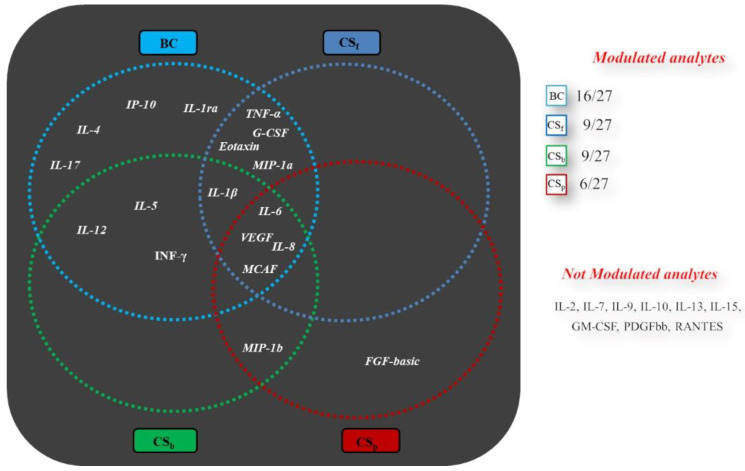
Schematic representation of biomarkers modulated by all the extractive and biofermentative chondroitins. The analysis was performed by multiplex assay. Fold Change considered vs. pCTRL: ≥1.20 or ≤0.80.

**Figure 2:**
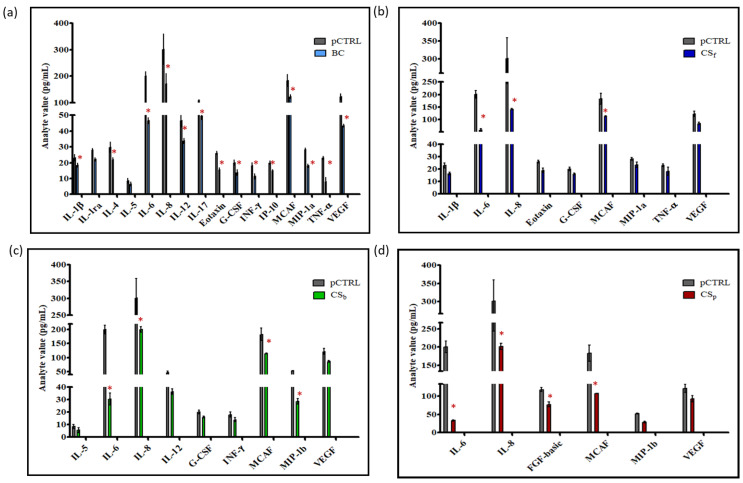
Multiplex assay. Only the analytes with a 0.8 ≥ fold change ≥ 1.2 (vs. pCTRL) were considered differentially modulated. * *p* < 0.05 vs. pCTRL. Data were divided as following: (**a**) pCTRL and BC, (**b**) pCTRL and CS_f_, (**c**) pCTRL and CS_b_, (**d**) pCTRL and CS_p_. pCTRL is the same for all the graphs.

It was previously shown that chondroitin treatments affect the extracellular secretion of important inflammation mediators in pathological synoviocytes [31]. However, the analyses were performed following long incubation times, especially considering the starvation conditions. Here, different chondroitins with respect to origin, sulfation and Mw (Table 1), were assayed for their action after 8 h of contact with cells, and compared to untreated pathological ones, to evaluate if this shorter time-frame could be sufficient to prompt a biological response. The modulated analytes are shown in Figure 1 and the specific measured values for cytokines, chemokines and growth factors in each treatment are reported in Figure 2a–d. In this context, CS_p_ treatment significantly modulated the secretion levels of 6 analytes out of the 27 assayed, while more factors were modulated by CS_f_ and CS_b_ (i.e., 9 out of 27) and BC (16 out of 27). Interestingly, all chondroitins, at the tested concentration, were able to significantly decrease pro-inflammatory biomarkers’ levels, such as IL-6, IL-8 and MCAF (vs. pCTRL, *p* < 0.05), but BC and CS_f_ treatments also downregulated TNF-α (Figure 2a and Figure 2b, respectively). In addition, BC treatment significantly (*p* < 0.05) reduced the secreted levels of two growth factors, G-CSF (FC 0.60) and VEGF (FC 0.66); similarly, CS_f_ significantly decreased G-CSF secretion (FC 0.78, *p* < 0.05) vs. pCTRL (Figure 2b). Overall, these results proved that short-time treatments based on both sulfated and unsulfated chondroitins are also effective in inducing a modulation of different OA-related mediators.

### 2.2. Western Blot Analyses

**Figure 3:**
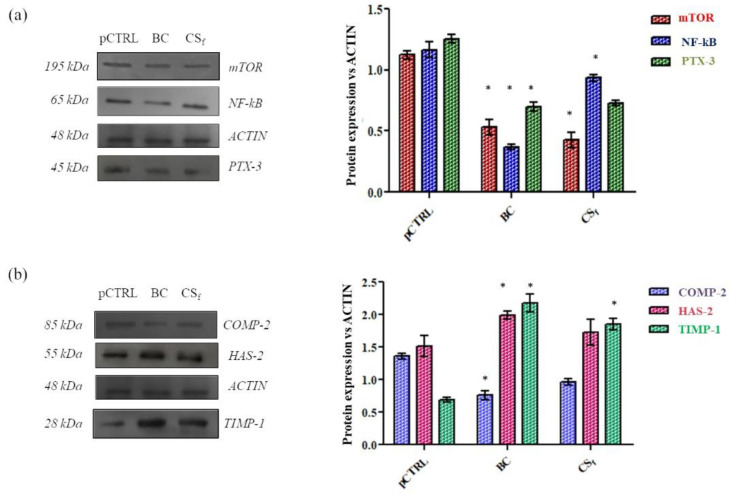
Western blot analysis in CS- and BC-treated OA synoviocytes. ACTIN was used as the loading control. (**a**) Biomarkers related to inflammatory process; (**b**) Biomarkers involved in remodeling and/or regeneration of cartilage. Relative changes of treated samples vs. pCTRL are reported as mean + SD. * *p* < 0.05.

We next analyzed the intra-cellular expression levels of well-recognized OA markers (i.e., COMP-2, NF-kB and PTX-3). Figure 3 displays that, after 24 h of treatment in a culture serum-free medium, BC and CS_f_ modulated the production of all analyzed biomarkers with respect to pCTRL. As expected, based on the source of isolated synoviocytes from OA synovial fluids, we detected basal levels of the proteins mTOR, COMP-2, NF-kB and PTX-3, thus confirming an ongoing inflammatory and degradative process. Both BC and CS_f_ treatments significantly (*p* < 0.05) downregulated mTOR (by 2.1 and 2.7 fold, respectively) and NF-kB (2.9 and 1.2 fold correspondingly) in comparison to pCTRL. In addition, PTX-3 protein levels were found to be significantly (*p* < 0.05) reduced, specifically, BC treatment was slightly more effective by 1.8 fold compared to untreated cells while CS_f_ reduced the expression of the same biomarker by about 1.7 fold (Figure 3a). Additionally, in COMP-2 negative modulation, BC also proved more active than CS_f_ with a decrease of 1.8 fold vs. pCTRL, in comparison to the 1.4 fold of the extractive one (Figure 3b). The degradation of the cartilage matrix is related to TIMP-1 expression since it may counteract the activity of metalloproteases. These analyses demonstrated the tested chondroitins, increased TIMP-1 expression, contributing to slow down the degradation process. In fact, TIMP-1 levels were higher (3.14 fold and 2.68 fold) (*p* < 0.05) in cells treated with BC and CS_f_ in comparison to pCTRL (Figure 3b). Finally, BC induced the increase in HAS-2 protein levels, thus supporting HA biosynthesis. HAS-2 protein expression resulted in being higher by about 1.3 fold for BC treatments, while CS_f_ treatments were similar to the pCTRL outcomes (about 0.90 fold).

### 2.3. Proteomic Analysis by High-Resolution nanoLC-MS/MS

**Figure 4:**
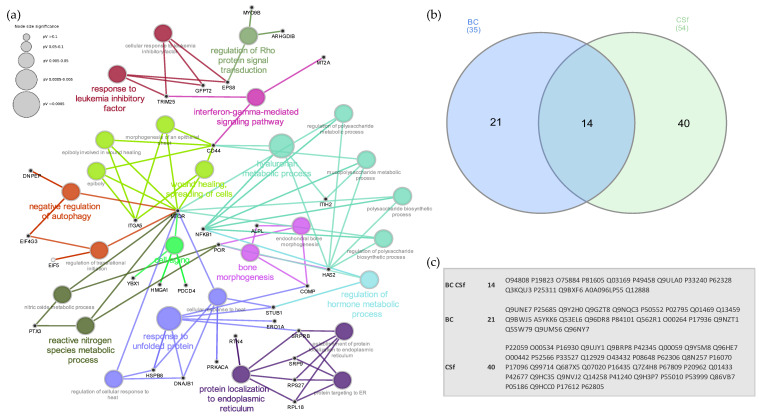
(**a**) Enriched GO network groups using ClueGO/CluePedia-based enrichment. A network view for GO Biological Processes of differentially regulated proteins following BC and CS_f_ treatments. Terms (each represented as node) are functionally grouped based on shared genes (kappa score ≥ 0.4) and are shown with different colors representing the class to which they belong. The specific players (proteins) of each node are highlighted with the respective gene name (in black). The size of the nodes indicates the degree of significance. Within each class, the most significant term (indicated with colored and bold characters) defines the name of the group. Ungrouped terms are not shown. (**b**) Venn diagram showing the overlapping of differentially expressed proteins after BC (blue) and CS_f_ (green) treatments. Details of the UniProt accession numbers within each subset of the Venn diagram are reported in (**c**).

To investigate the effects of BC and CS_f_ treatments on the overall protein profile at the intracellular level, comparative proteomic analyses were carried out by applying a quantitative nanoLC-MS/MS TMT isobaric labelling-based approach on treated and untreated starved human primary-synoviocytes. Using the Proteome Discoverer proteomics software package, we compared protein abundances of CS_f_-/BC-treated OA synoviocytes with respect to untreated cells. In particular, for each treatment replicate, we required a minimum of two replicates and at least two peptides per protein in at least one out of three conditions for the identification to be considered reliable. According to these criteria, a total of 1558 proteins were identified across the three conditions (Table S1 (Supplementary Materials)). From this list, we then extracted a subset of 75 proteins whose expression levels changed 1.2-fold or more (in any direction) in both (Table 2) or one (Table 3) of the treatment conditions with respect to untreated OA synoviocytes. Bioinformatics enrichment analysis performed for the biological process GO category (Figure 4a) revealed that a significant number of identified proteins are involved in processes related to hyaluronan metabolic process (17.9%), wound healing (14.3%), response to unfolded protein/protein localization to endoplasmic reticulum (10.7%) and bone morphogenesis (7.1%). Proteins related to the reactive nitrogen species metabolic process (7.1%), response to leukemia inhibitory factor (7.1%) and regulation of autophagy (7.1%) were also identified. A small fraction (about 4.9%) of identified proteins was found differentially expressed (0.8 ≥ fold change ≥ 1.2) across the cells treated with either CS_f_ or BC compared to untreated cells (Table 2 and Table 3). We then investigated if there was a correlation of responses to different chondroitin treatments by comparing differentially expressed proteins to different treatments. We found that a specific subset of 14 proteins was commonly differentially expressed in both treatments (Figure 4b). Despite this partial similar response to the different treatments, for some differentially expressed proteins, a distinctive response of OA synoviocytes to BC and CS was also observed thus suggesting the modulation of both common and distinct responses to chondroitin treatments. The heatmap representing the log_2_ fold-change values of differentially expressed (0.8 ≥ FC ≥ 1.2) in CS_f_- and/or BC-treated vs. pCTRL synoviocytes identified by high-resolution LC-MS/MS was obtained and is provided as Figure S1 (Supplementary Materials).

## 3. Discussion

Because resolutive treatments for OA pathology are not currently available, often patients need to use anti-inflammatory drugs or cortisone to reduce pain. Some international Guidelines suggest the use of chondroitin sulfate in oral formulations in the early stage of OA. GAGs-based intra-articular injections of specifically formulated gels may improve functionality through the viscoelastic features, reduce ache and, possibly, in the short term, counteract pathology progression [36,37]. Recently, different scientific approaches are focused on the identification of key biomarkers to evaluate the potential beneficial effects of different treatments. Among these, mass spectrometry approaches play a key role [17,24,38,39]. Indeed, proteomic studies aimed at investigating the response of joint-derived cells to different treatments such as HA, GlcN, extractive CS and, more recently, the recently available high-purity biofermentative unsulfated chondroitin, have been increasing in the last few years. The scientific literature also reports data about the secretome of chondrocyte in in vitro cultures [40,41]. It has been demonstrated that the pro-inflammatory mediators secreted by synovial cells play a key role in the chondrocytes’ activation process by increasing the production of several cytokines and chemokines [42]. For this reason, the response of pathological synoviocytes, at both secretome and proteome levels, following specific GAGs treatments (considering that some of those are injective) is necessary to support the effectiveness of potential OA therapies based on these biopolymers toward the relief of symptoms and movement [6,40]. Recent in vitro and in vivo studies showed a positive effect of CS in human chondrocytes and damaged cartilage [43,44]. The well-accepted scientific hypothesis is the involvement of CS in the inhibition of NF-kB signaling and increasing HA biosynthesis. In this way, CS may reduce the pain through the modulation of the bradykinin (BK) system that is related to the release of IL-6 and IL-8 via p38MAPK and NF-kB activation in synoviocytes and chondrocytes [44]. Most CS used for OA symptomatic management is derived from animal sources such as bovine trachea or porcine (ear and nose) tissues and are namely terrestrial CS. However, fish-derived CS has been also obtained and commercialized for two decades. Specifically, the shark fin-derived product is regularly available, under certification that the protected species are not among the ones sacrificed within the manufacturing process. Terrestrial and marine CS have different biochemical features, presenting diverse molecular weight and sulfation pattern [45,46]. It has been argued that the combination between a specific sequence of disaccharides and the sulfation grade is responsible for a particular biological activity. Accordingly, Pomin et al. 2019 [45] clearly explained that marine CS may be considered a very interesting biomolecule for the presence of sulfation patterns that are not found in terrestrial animals. Marine CS showed anti-inflammatory activities and improved the mechanical performance of cartilage-engineered constructs [46]. Moreover, different studies demonstrated that marine CS has important effects on in vitro chondrocytes’ proliferation and differentiation but the scientific knowledge about its application in OA management is less diffused than the terrestrial CS [45,47]. In addition, very recent studies demonstrated that both marine CS and BC are also related to the differentiation of mesenchymal cells towards the chondrocyte phenotype when added to the culture medium or as a component of chemically modified gelatin-based scaffolds [48,49]. We recently reported that CS_f_ and BC significantly affect the synoviocytes secretome and that BC is able (both alone and coupled to HA) to reduce the inflammatory process in this OA in vitro model [30,31]. Despite the long tradition of using both pharma- and food-grade CS, the specific biological mechanism involved still needs to be fully unraveled. Due to being the polymer dimension and charge density often responsible for interactions with cells and other macromolecules of the extracellular matrix network, the studies aimed at characterizing the bioactivity of the recently available unsulfated BC are of remarkable scientific interest. Recent published data showed that chondroitins (both marine and biofermentative) were able to modulate the secretion of many OA-correlated biomarkers after 48 h of treatment [31]. In the present study, the quantification of these latter (for a total of 27) was performed after a shorter incubation time (8 h) in order to also verify the effectiveness of chondroitin in this condition. The outcomes displayed that the biomarkers strongly involved in the inflammatory cellular response, such as IL-6, IL-8 and MCAF, were significantly reduced by BC and all CS treatments, thus highlighting a generally positive anti-inflammatory effect [50,51]. The analysis of specific inflammatory response-correlated mediators (mTOR, NF-kB, PTX-3 and COMP-2) confirmed the ability of both CS_f_ and BC to affect these proteins [8,52,53,54]. Furthermore, CS_f_ and BC showed similar effectiveness in increasing TIMP-1 levels; this latter is known to contribute to attenuating ECM degradation through the inhibition of metalloproteases action [55]. Another selected biomarker was HAS-2, since this enzyme is involved in HA biosynthesis, which in turn affects the rheological features of synovial fluid, and thus the lubrication and shock absorption ability. HA content and size in synovial fluid may affect joint movement, thus marked upregulation of HAS-2 has to be considered desirable [12,13]. Our study provides new information on the effects of sulfated and unsulfated chondroitins on human OA synoviocytes proteomes by means of a Tandem Mass Tag (TMT)-based high-resolution LC-MS/MS approach. Both treatments affected the expression levels of proteins involved in important biological pathways converging on the mTOR and NF-kB signaling, affecting hyaluronan metabolism and the response to reactive nitrogen species metabolic processes. These findings are coherent with the literature, identifying the PI3K/AKT/mTOR signaling pathway as essential for the metabolism of joint tissues. The mTOR signaling pathway has been related to the development of OA, cartilage degradation, subchondral bone dysfunction and synovial inflammation [56]. Proteins related to the response to leukemia inhibitory factor (LIF) and regulation of autophagy were also differentially expressed following chondroitin treatments. In this respect, both the role of LIF and autophagy are critical among OA progression [56,57]. Furthermore, as previously observed for secretome profiles [31], despite similarities among BC and CS_f_ treatments, for several up- and downregulated proteins a different regulation trend was also observed, suggesting a certain degree of specificity in this specific experimental set-up. From a production point of view, it should be considered that BC is produced by bacterial fermentation, thus overcoming any issues related to the extraction source, such as safety concerns and/or the environmental impact of the manufacturing process. Overall, our results showed that unsulfated chondroitin bioactivity resembles and is comparable to extractive CS, thus increasing our knowledge about biomarkers and treatments that may be supportive in the clinical management of OA-affected patients.

## 4. Materials and Methods

### 4.1. Preparation of CS and BC Solutions

BC was produced in our laboratories through a patented fermentation process using EcK4r3, a specific recombinant strain. The experimental protocols followed were previously reported [32,33]. The obtained BC (MW; 35 ± 3 kDa) had a purity of 95 ± 5% and a low endotoxin content was confirmed through Limulus test (EU/mg < 0.05). The CSs used during this experimental work were provided, in the framework of a collaboration with BioTekNet Scpa, by the IBSA group and were obtained from diverse animal sources, pig (CS_p_), bovine (CS_b_) and fish, specifically shark (CS_f_), with high purity (95 ± 5%), and a very low endotoxin content (0.1 EU/mg). The CSs are manufactured according to ICHQ7 and EudraLex volume 4 part II. Furthermore, the latter were obtained from cartilages collected from species that are not mentioned in the list of protected species of CITIES (the Convention of International Trade in Endangered Species of wild fauna and flora), under the responsibility of the IBSA Chinese subsidiary. BC and CSs were dissolved in Phosphate-Buffered Saline (PBS, pH 7.2; Lonza, Milan, Italy) at a concentration of 16 mg/mL. The pH and osmolality of the obtained solutions were measured to perform experimental tests under physiological conditions (i.e., pH 7.0 ± 0.1 and osmolality 300 mOsm); later they were sterilized by autoclave (1 bar, 121 °C for, 20 min). Lastly, BC- and CS-based gels were diluted 1:5 in a specific culture medium (Dulbecco’s modified Eagle’s medium, Gibco DMEM, without fetal bovine serum Gibco FBS; Fisher Scientific Italia, Milan, Italy) and microfiltered (0.22 µm; Millipore, Milan, Italy).

### 4.2. OA In Vitro Model and Chondroitin-Based Treatments

As previously reported, human primary articular synoviocytes were isolated from the synovial fluids of OA-affected patients, during knee joint replacement surgical procedures. These operations were performed at the Orthopedics and Traumatology Department of the University Federico II of Naples. All patients gave informed consent, and the procedures were approved by the Internal Ethical Committee. After the isolation, the cell phenotype was confirmed using the protocols previously established in our laboratories [30]. For the cell treatments, we followed the starvation protocol described by Russo et al. 2020 [31] with slight changes. Specifically, the cells were cultured in serum-free medium with or without BC (3.2 mg/mL), and CSs (3.2 mg/mL) for 8 h or 24 h in a standard 24 well culture plate or in a 25 mm^2^ flask (Falcon DB).

### 4.3. Bioplex Assay

A 27-plex immunoassay panel based on xMAP technology [58] was used to evaluate the modulation of specific pro-inflammatory and anti-inflammatory cytokines, chemokines and growth factors (Bio-Rad Laboratories s.r.l., Milan, Italy) after the treatments of primary synoviocytes with BC or CSs. After 8 h of treatments, the culture medium for each sample was withdrawn, centrifuged (1500 rpm, 7 min) and used to perform the bioplex assay. The specific panel permitted to evaluate the pro-inflammatory cytokines: IL-1β, IL-1ra, IL-2, IL-5, IL-6, IL-7, IL-9, IL-12, IL-13, IL-15, IL-17, INF-γ, TNF-α; anti-inflammatory cytokines: IL-4, IL-10; growth factors: G-CSF, GM-CSF, FGFbasic, PDGF-bb, VEGF and chemokines: IL-8, MCAF, MIP-1a, MIP-1b, RANTES, Eotaxin, IP-10. The technique was based on the use of magnetic beads labeled with red and infrared fluorophores covered with specific antibodies allowing the simultaneous evaluation of multiple target analytes within a single sample [31]. The binding of each particle with the target analyte detection was performed with a biotinylated antibody and phycoerythrin-conjugated streptavidin. The procedures were carried out following the manufacturer’s instructions using a Bio-Plex array reader (Luminex, Austin), TXAnalyte concentrations (pg/mL) were assessed by a standard curve according to the manufacturer’s protocol. The biological mediators were considered differentially modulated when CS_f_- or BC-based treatments induced a decrease and/or increase in the biomarker with a ratio 0.8 ≥ fold change ≥ 1.2 in comparison to untreated cells.

### 4.4. Western Blot Analyses

For Western Blot analyses, 1.0 × 10^5^ cells were seeded in a 25 mm^2^ flask and treated with BC or CS_f_ for 24 h. Following treatments, cells were harvested and lysed by a Radio-Immunoprecipitation Assay buffer (RIPA buffer 1×) (Cell Signaling Technology, Danvers, MA, USA). Protein concentration for each sample was determined using the Bradford method [59]. Western blotting analyses were performed as previously described [60]. Briefly, aliquots of intracellular proteins (30 µg) were resolved by electrophoresis on 10% SDS-PAGE and transferred to a nitrocellulose membrane (GE, Amersham, Chicago, IL, USA). Equivalent loadings were verified by Ponceau Red (Sigma-Aldrich, St. Louis, MO, USA) staining after transfer. Then, the membrane was blocked by 5% skimmed milk in Tris-buffered saline and 0.05% Tween-20 (TTBS) for 30 min. Primary antibodies to detect mTOR (Cell Signaling Technology, Danvers, MA, USA), COMP-2 (Abcam, Cambridge, UK), NF-kB (Santa Cruz Biotechnology, Dallas, TX, USA), HAS-2 (Santa Cruz Biotechnology, Dallas, TX USA), PTX-3 (Santa Cruz Biotechnology, Dallas, TX, USA) and TIMP-1 (Santa Cruz Biotechnology, Dallas, TX, USA) were diluted 1:500, 1:250, 1:200, 1:200, 1:100, respectively, and incubated overnight at 4 °C. After that, the membrane was extensively washed using TTBS and immunoreactive bands were detected using chemiluminescence suitable horseradish peroxidase-conjugated secondary antibodies diluted 1:5000 (Santa Cruz Biotechnology, Dallas, TX, USA). Following incubation for 2 h at room temperature, signals detection was performed with an ECL system (Merck Millipore, Burlington, MA, USA). Anti-Actin antibody (Santa Cruz Biotechnology, Dallas, TX, USA) was diluted 1:1000 and used to normalize the protein levels of each analyzed biomarker. A semi-quantitative analysis of protein expressions was carried out by using the ImageJ program according to the manufacturer’s protocol.

### 4.5. Data Analysis

Data are expressed as mean ± standard deviation (SD). The statistical significance of the differences between BC- and CS-treated synoviocytes samples vs. pCTRL was determined using a two-tailed *t*-test, considering *p* values < 0.05. As explained in the following, for the proteomic analyses the statistical significance was set to 0.05 (*p* ≤ 0.05), and the Bonferroni test was used to correct the *p*-value.

### 4.6. Sample Preparation for Proteomic Analyses

For proteomic analysis, human primary articular synoviocytes treated and untreated with BC or CS_f_ as described above were lysed in ice-cold lysis buffer (100 mM Triethylammonium bicarbonate TEAB, SDS 1%) and disrupted by two cycles of sonication at a 20% amplitude for 30 sec on ice. Lysates were cleared by centrifugation at 16,000× *g* for 15 min at 4 °C. Supernatants were transferred into new tubes and treated with 1 Unit of RQ1 DNase (Promega, Milan, Italy) for 1 h at room temperature. Protein concentration was determined by using the Pierce BCA Protein assay kit (Thermo Scientific, Milan, Italy). For each condition, equal amounts of proteins (100 µg in 100 µL of 100 mM TEAB) were reduced with 10 mM Tris-(2-carboxyethyl)-phosphine (TCEP) for 1 h at 55 °C and alkylated with 18 mM iodoacetamide by incubating samples for 30 min at room temperature in the dark. Proteins were then precipitated overnight by adding six volumes of pre-chilled acetone. Following centrifugation at 8000× *g* for 10 min at 4 °C, protein pellets were resuspended in 100 µL of 100 mM TEAB and digested overnight with MS-grade trypsin (Thermo Scientific, USA) at an enzyme/substrate ratio of 1:40 at 37 °C. The resulting peptide mixtures were chemically labeled with the TMT isobaric tags as previously reported [61,62] using the 128C, 127N and 126 tags for the BC- and CS_f_-treated and untreated cells, respectively. Briefly, 0.8 mg of TMT reagents in 41 µL of anhydrous acetonitrile were added to each sample. The reaction proceeded for 1 h and then was quenched for 15 min with hydroxylamine to a final concentration of 0.3% (*w*/*w*). The two samples were then mixed at equal amounts and diluted in 0.1% TFA/2% CH_3_CN to a final concentration of 0.5 µg/µL for LC-MS analyses.

### 4.7. High-Resolution nanoLC−Tandem Mass Spectrometry

Aliquots of TMT-labeled samples (2.5 µg) were analyzed in triplicate by high-resolution nanoLC−Tandem Mass Spectrometry using a Q-Exactive Orbitrap mass spectrometer equipped with an EASY-Spray nano-electrospray ion source (Thermo Fisher Scientific, Germany) and coupled to a Thermo Scientific Dionex UltiMate 3000RSLC nanosystem (Thermo Fisher Scientific) as previously reported [63]. The solvent composition was 0.1% formic acid in water (solvent A) and 0.1% formic acid in acetonitrile (solvent B). Peptides were loaded on a trapping PepMap™ 100 μ Cartridge Column C18 (300 μm × 0.5 cm, 5 μm, 100 Å) and desalted with solvent A for 3 min at a flow rate of 10 μL/min. After trapping, the eluted peptides were separated on an EASY-Spray analytical column (50 cm × 75 μm ID PepMap RSLC C18, 3 μm, 100 Angstrom), heated at 35 °C, at a flow rate of 300 nL/min applying the following gradient: 5% B for 3 min; from 5% to 27.5% B in 222 min; from 27.5% to 40% B in 10 min; from 40% to 95% B in 1 min. Washing (95% B for 4 min) and re-equilibration (5% B for 24 min) steps were always included at the end of the gradient. Eluting peptides were analyzed on the Q-Exactive mass spectrometer operating in positive polarity mode with a capillary temperature of 280 °C and a potential of 1.9 kV applied to the capillary probe. Full MS survey scan resolution was set to 70,000 with an automatic gain control (AGC) target value of 3 × 10^6^ for a scan range of 375–1500 *m*/*z* and maximum ion injection time (IT) of 60 ms. The mass (*m*/*z*) 445.12003 was used as lock mass. A data-dependent top 12 method was operated, during which high-energy collisional dissociation (HCD) spectra were obtained at a 35000 MS2 resolution with an AGC target of 1 × 10^5^ for a scan range of 200–2000 *m*/*z*, maximum IT of 120 ms, 1.6 *m*/*z* isolation width and normalized collisional energy (NCE) of 32. Precursor ions targeted for HCD were dynamically excluded for 30 s. Full scans and Orbitrap MS/MS scans were acquired in profile mode, whereas ion trap mass spectra were acquired in centroid mode. Charge state recognition was enabled by excluding unassigned and 1, 7, 8, >8 charged states. All data were acquired with the Xcalibur 3.1 software (Thermo-Fisher Scientific).

### 4.8. Protein Identification and Quantitation

For data processing, the acquired raw files were analyzed with the Thermo Scientific Proteome Discoverer 2.4 software (Thermo Fisher Scientific) using the SEQUEST HT search engine. The HCD MS/MS spectra were searched against the Homo sapiens database (release 2019_11, 20,380 entries) assuming trypsin (Full) as the digestion enzyme and two allowed numbers of missed cleavage sites. Mass tolerances were set to 10 ppm and 0.02 Da for precursor and fragment ions, respectively. Oxidation of methionine (+15.995 Da) was set as a dynamic modification. Carbamidomethylation of cysteine (+57.021 Da) and the TMT label on lysines and the N-terminus (229.1629) were set as static modifications. False discovery rates (FDRs) for peptide spectral matches (PSMs) were calculated and filtered using the Percolator node in Proteome Discoverer that was run with the following settings: Maximum Delta Cn 0.05, a strict target FDR of 0.01, a relaxed target FDR of 0.05 and validation based on q-value. Protein identifications were accepted when the protein FDR was below 1% and when present in at least two out of three replicate injections with at least two peptides. Fold Change (FC) thresholds of proteins identified in BC and CS_f_-treated vs. untreated cells were set at ±1.2.

### 4.9. Bioinformatic Analyses

The clustered heatmap of the proteins differentially expressed in at least one out of two treatments was generated by using the CIMminer freely available web-server tool (http://discover.nci.nih.gov/cimminer/ accessed on 5 December 2021) with unsupervised clustering set on raws only with the following parameters: average linkage, Euclidean distance, and quantile binning. The ClueGO v2.5.8 + CluePedia v1.5.4, a Cytoscape v3.8.2 plug-in, was used to visualize the non-redundant biological process GO terms of proteins up- and downregulated following BC or CS_f_ treatments in functionally organized networks reflecting the relationships between the biological terms based on the similarity of their linked gene/proteins [40]. Biomarkers related to inflammation and selected for Western blot analyses (i.e., mTOR, COMP-2, NF-kB, HAS-2, TIMP-1 and PTX-3) were included in the input list to verify the occurrence of specific connections between target analytes and differentially expressed proteins. For the enrichment of biological terms and groups, the two-sided (Enrichment/Depletion) tests based on the hypergeometric distribution was used. The statistical significance was set to 0.05 (*p* ≤ 0.05), and the Bonferroni step down adjustment was used to correct the *p*-value for the terms and the groups created by ClueGO. To diminish the redundancy of the terms shared by similar associated proteins, the network specificity was set to Medium, and the GO Term grouping option was selected, which allows the maintenance of the most representative parent or child term. The parameters were: kappa score threshold set to 0.4; Leading Group term based on: Highest Significance; % gene for Group Merge: 50; % Terms for Group Merge: 50.

## 5. Conclusions

This study emphasizes the importance of robust in vitro models, resembling OA pathology, to compare chondroitin of different origins, with an extensive molecular fingerprint, based on multiplex assay and quantitative proteomic approaches, to shed light on the peculiar biological features of these intriguing macromolecules. Considering that chondroitin-based treatments have more and more relevance in the management of osteoarthritis, which reports an increasing number of patients every year, a better understanding of the biochemical characteristics of the animal source and biotechnological chondroitins, also in relation to the purity, molecular weight and sulfation pattern, may help in assessing their therapeutic use, either contemporary to other pharmacological treatments or as a main drug to counteract joint-disease progression and patient pain.

## Figures and Tables

**Table 1:** Hydrodynamic characterization of chondroitins through Size Exclusion Chromatography coupled to triple array detector (SEC-TDA), molecular weight, polydispersity and purity evaluation.

Chondroitin	Mw (kDa)	Mw/Mn	Recovery
Chondroitin fish	32.65	1.18	>96%
Chondroitin bovine	18.0	1.7	>96%
Chondroitin pig	17.7	1.24	99.3%
Chondroitin biofermentative	31.5	1.22	>96%

**Table 2:** Proteins differentially expressed (0.8 ≥ FC ≥ 1.2) in both CS_f_- and BC-treated vs. pCTRL synoviocytes identified by high-resolution LC-MS/MS.

Accession	Gene Name	Description	CS_f_ vs. pCTRL	BC vs. pCTRL
O94808	GFPT2	Glutamine—fructose-6-phosphate aminotransferase (isomerizing) 2	1.2	1.4
Q3KQU3	MAP7D1	MAP7 domain-containing protein 1	1.2	1.4
P62328	TMSB4X	Thymosin beta-4	1.2	1.3
Q12888	TP53BP1	TP53-binding protein 1	1.3	1.3
O75884	RBBP9	Serine hydrolase RBBP9	1.2	1.3
Q9ULA0	DNPEP	Aspartyl aminopeptidase	1.2	1.3
P25311	AZGP1	Zinc-alpha-2-glycoprotein	0.8	1.3
P33240	CSTF2	Cleavage stimulation factor subunit 2	1.2	1.2
Q9BXF6	RAB11FIP5	Rab11 family-interacting protein 5	1.3	1.2
Q03169	TNFAIP2	Tumor necrosis factor alpha-induced protein 2	1.3	1.2
P49458	SRP9	Signal recognition particle 9 kDa protein	1.2	1.2
A0A096LP55	UQCRHL	Cytochrome b-c1 complex subunit 6-like, mitochondrial	1.2	1.2
P19823	ITIH2	Inter-alpha-trypsin inhibitor heavy chain H2	0.6	0.8
P81605	DCD	Dermcidin	0.5	0.7
Q96NY7	CLIC6	Chloride intracellular channel protein 6	1.2	1.3
Q53EL6	PDCD4	Programmed cell death protein 4	1.2	1.3
Q9BWJ5	SF3B5	Splicing factor 3B subunit 5	1.2	1.3
A5YKK6	CNOT1	CCR4-NOT transcription complex subunit 1	1.2	1.3
O00264	PGRMC1	Membrane-associated progesterone receptor component 1	1.2	1.2
P62306	SNRPF	Small nuclear ribonucleoprotein F	1.3	1.2
P22059	OSBP	Oxysterol-binding protein 1	1.2	1.2
Q9BRP8	PYM1	Partner of Y14 and mago	1.3	1.2
O43432	EIF4G3	Eukaryotic translation initiation factor 4 gamma 3	1.2	1.2

**Table 3:** Proteins differentially regulated (0.8 ≥ FC ≥ 1.2) in CS_f_- or BC-treated vs. pCTRL synoviocytes identified by high-resolution LC-MS/MS.

Accession	Gene Name	Description	CS_f_ vs. pCTRL	BC vs. pCTRL
Q96HE7	ERO1A	ERO1-like protein alpha	1.2	1.1
Q12929	EPS8	Epidermal growth factor receptor kinase substrate 8	1.3	1.1
Q99714	HSD17B10	3-hydroxyacyl-CoA dehydrogenase type-2	1.2	1.1
O00442	RTCA	RNA 3′-terminal phosphate cyclase	1.3	1.1
Q9H3P7	ACBD3	Golgi resident protein GCP60	1.4	1.1
P55010	EIF5	Eukaryotic translation initiation factor 5	1.2	1.1
Q14258	TRIM25	E3 ubiquitin/ISG15 ligase TRIM25	1.3	1.1
Q00059	TFAM	Transcription factor A, mitochondrial	1.2	1.1
P67809	YBX1	Y-box-binding protein 1	1.2	1.1
Q9HCC0	MCCC2	Methylcrotonoyl-CoA carboxylase beta chain, mitochondrial	1.2	1.1
Q01433	AMPD2	AMP deaminase 2	1.2	1.1
O00534	VWA5A	von Willebrand factor A domain-containing protein 5A	1.3	1.1
P33527	ABCC1	Multidrug resistance-associated protein 1	1.2	1.1
P17096	HMGA1	High mobility group protein HMG-I/HMG-Y	0.8	1.1
P42345	MTOR	Serine/threonine-protein kinase mTOR	1.2	1.1
P16435	POR	NADPH--cytochrome P450 reductase	1.3	1.1
Q7Z4H8	POGLUT3	Protein O-glucosyltransferase 3	1.2	1.1
P62805	H4	Histone H4	1.3	1.1
P53999	SUB1	Activated RNA polymerase II transcriptional coactivator p15	1.2	1.0
P17612	PRKACA	cAMP-dependent protein kinase catalytic subunit alpha	1.2	1.0
Q9UJY1	HSPB8	Heat shock protein beta-8	1.2	1.0
P20962	PTMS	Parathymosin	0.8	1.0
Q9Y5M8	SRPRB	Signal recognition particle receptor subunit beta	1.2	1.0
P05186	ALPL	Alkaline phosphatase, tissue-nonspecific isozyme	1.2	1.0
Q07020	RPL18	60S ribosomal protein L18	1.2	1.0
P42677	RPS27	40S ribosomal protein S27	1.2	1.0
Q687X5	STEAP4	Metalloreductase STEAP4	1.2	1.0
Q8N257	H2BU1	Histone H2B type 3-B	1.2	1.0
Q86VB7	CD163	Scavenger receptor cysteine-rich type 1 protein M130	0.7	1.0
Q9NVJ2	ARL8B	ADP-ribosylation factor-like protein 8B	1.2	1.0
P16930	FAH	Fumarylacetoacetase	1.2	1.0
P08648	ITGA5	Integrin alpha-5	0.7	1.0
P16070	CD44	CD44 antigen	0.8	1.0
P52566	ARHGDIB	Rho GDP-dissociation inhibitor 2	0.8	0.9
P41240	CSK	Tyrosine-protein kinase CSK	0.8	0.9
Q9HC35	EML4	Echinoderm microtubule-associated protein-like 4	0.8	0.9
Q96DR8	MUCL1	Mucin-like protein 1	1.0	6.7
Q9GZT8	NIF3L1	NIF3-like protein 1	1.1	1.8
Q9NZT1	CALML5	Calmodulin-like protein 5	1.1	1.7
Q9NQC3	RTN4	Reticulon-4	1.1	1.6
P84101	SERF2	Small EDRK-rich factor 2	1.0	1.3
P02795	MT2A	Metallothionein-2	1.0	1.3
P17936	IGFBP3	Insulin-like growth factor-binding protein 3	1.1	1.3
P50552	VASP	Vasodilator-stimulated phosphoprotein	1.0	1.3
Q9UMS6	SYNPO2	Synaptopodin-2	1.1	1.3
Q5SW79	CEP170	Centrosomal protein of 170 kDa	1.1	1.3
Q13459	MYO9B	Unconventional myosin-IXb	1.1	1.2
Q9UNE7	STUB1	E3 ubiquitin-protein ligase CHIP	1.1	1.2
P25685	DNAJB1	DnaJ homolog subfamily B member 1	0.9	1.2
Q9Y2H0	DLGAP4	Disks large-associated protein 4	1.0	1.2
Q01469	FABP5	Fatty acid-binding protein 5	0.9	1.2
Q562R1	ACTBL2	Beta-actin-like protein 2	0.9	0.8

## Data Availability

All data critical for reader understanding and outcomes’ discussion are reported within the manuscript or as part of the Supplementary Materials. Additional raw data are available from the corresponding author upon request.

## Supplementary Materials

The following supporting information can be downloaded at: https://www.mdpi.com/article/10.3390/ijms232415865/s1.
