# Supplementary figures and images for: Molecular Fingerprint of Human Pathological Synoviocytes in Response to Extractive Sulfated and Biofermentative Unsulfated Chondroitins
Source: Int J Mol Sci. 2022 Dec 14;23(24):15865. doi: 10.3390/ijms232415865 (PMC9784855; doi:10.3390/ijms232415865)

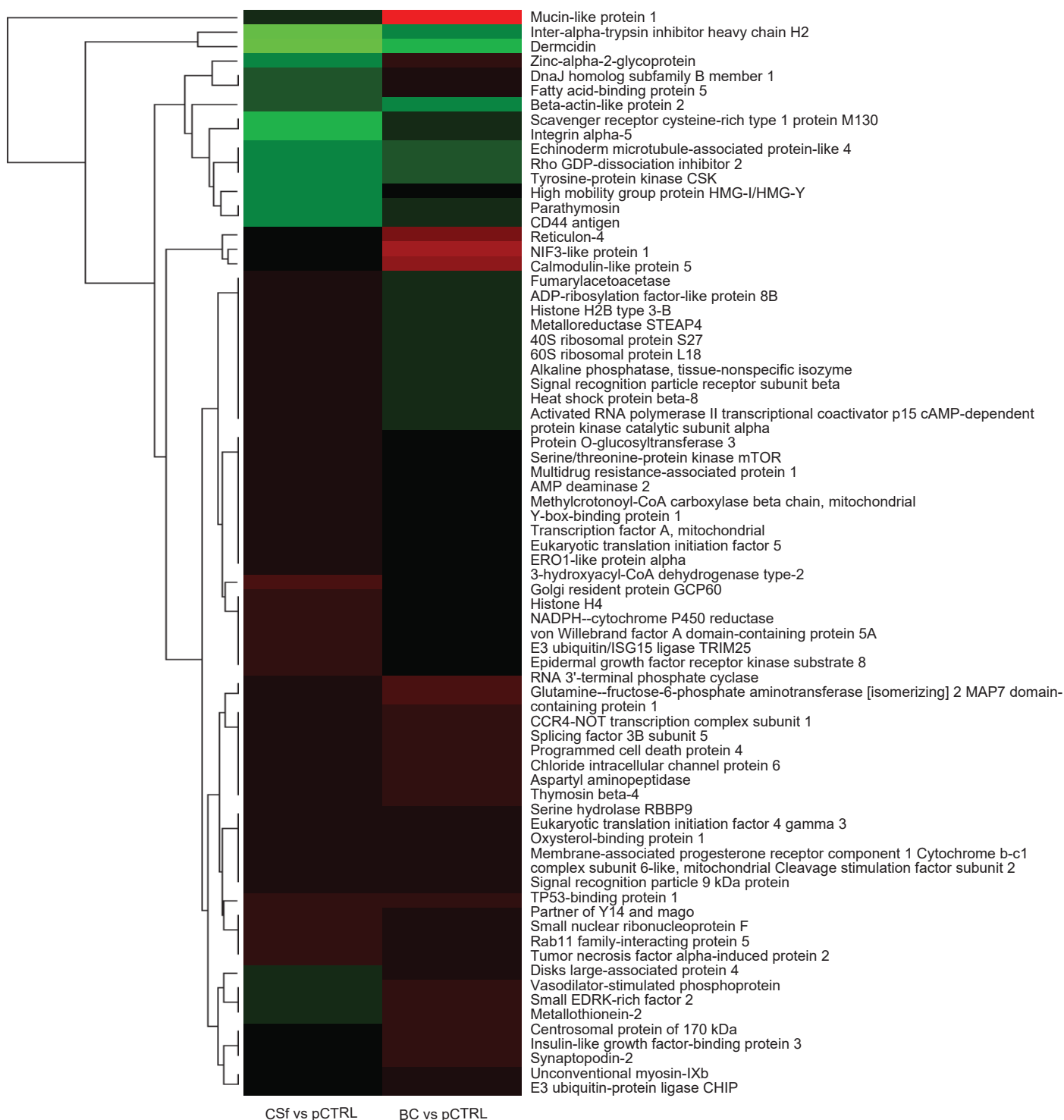

Supplement: Supplementary file 1 [file ijms-23-15865-s001.zip › ijms-1993002-Supplementary Figure S1.pdf]
